# Resolving the Role of Actoymyosin Contractility in Cell Microrheology

**DOI:** 10.1371/journal.pone.0007054

**Published:** 2009-09-16

**Authors:** Christopher M. Hale, Sean X. Sun, Denis Wirtz

**Affiliations:** 1 Department of Chemical and Biomolecular Engineering, The Johns Hopkins University, Baltimore, Maryland, United States of America; 2 Department of Mechanical Engineering, The Johns Hopkins University, Baltimore, Maryland, United States of America; 3 Institute for NanoBioTechnology, The Johns Hopkins University, Baltimore, Maryland, United States of America; Dalhousie University, Canada

## Abstract

Einstein's original description of Brownian motion established a direct relationship between thermally-excited random forces and the transport properties of a submicron particle in a viscous liquid. Recent work based on reconstituted actin filament networks suggests that nonthermal forces driven by the motor protein myosin II can induce large non-equilibrium fluctuations that dominate the motion of particles in cytoskeletal networks. Here, using high-resolution particle tracking, we find that thermal forces, not myosin-induced fluctuating forces, drive the motion of submicron particles embedded in the cytoskeleton of living cells. These results resolve the roles of myosin II and contractile actomyosin structures in the motion of nanoparticles lodged in the cytoplasm, reveal the biphasic mechanical architecture of adherent cells—stiff contractile stress fibers interdigitating in a network at the cell cortex and a soft actin meshwork in the body of the cell, validate the method of particle tracking-microrheology, and reconcile seemingly disparate atomic force microscopy (AFM) and particle-tracking microrheology measurements of living cells.

## Introduction

At long time scales, a submicron bead immersed in a viscous liquid is subjected to two dominant forces: a small random thermally-induced force and an equal and opposite frictional viscous force. The random force is induced by the dynamic random bombardment of the molecules of the suspending liquid on the surface of the bead. The frictional force is proportional to the velocity of the bead and its friction coefficient. As a result of these two forces, a submicron bead in a viscous liquid undergoes Brownian motion whereby it moves continuously in a random fashion, instantaneously losing directional memory. Einstein first established a theoretical description of Brownian motion and showed that the diffusion coefficient 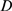 of a submicron bead is related to the viscosity of the suspending liquid, 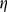, through the thermal energy 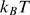 as 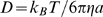, where 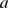 is the radius of the bead [1]. This simple relationship describes fundamental physics that relate energy, transport, and size; this relationship also indirectly confirmed the existence of atoms and molecules [1], [2].

Recently, Mizuno *et al.* [3] investigated the validity of the fluctuation-dissipation (FD) theorem, a generalization of Einstein's equation of Brownian motion, in an *in vitro* network comprised of purified actin filaments with embedded force-generating myosin II motors. While the FD theorem can be applied to tracer particles whose motion is induced by the random thermal force that causes conventional Brownian motion [4], forces of nonthermal origin [5], [6], [7] can cause violations of the FD theorem. In non-muscle cells, myosin II molecules form short filaments that promote the formation of actin filament bundles through F-actin binding domains and turn these bundles into contractile, bipolar, muscle-like structures. In the absence of myosin, beads inside reconstituted F-actin networks undergo Brownian motion [8], [9], albeit their displacements are elastically restricted compared to beads in buffer. According to Mizuno *et al.*, in the presence of activated myosin II, the amplitude of the fluctuations of embedded beads is increased by orders of magnitude, especially at long time scales [3].

These authors further showed that the addition of myosin II to an F-actin system is akin to a 50-200-fold increase in the temperature of the system compared to an inactivated myosin/F-actin system. This implies that tension-generating motor proteins generate giant fluctuations that are much larger than fluctuations powered by thermal energy, 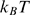. More importantly, the authors concluded that the FD theorem is violated strictly due to the contractility of the actomyosin system. A consequence of this provocative result is that the basic assumption of the commonly used approach of particle tracking microrheology [10], [11], [12] used to probe the mechanical properties of cytoplasm—that beads inside cells are only driven by thermal forces—is incorrect, for microrheology would greatly underestimate the elasticity of the cytoskeleton in living cells.

While a reductionist approach can often help our understanding of cytoskeleton biophysics [13], the molecular composition, density, and organization of a reconstituted actin filament network composed of purified actin and myosin are vastly different from those of cytoskeletal networks in living cells [14]. Therefore, it is essential to assess the impact of actomyosin contractility on the motion of tracer particles embedded in the cytoskeleton of the cell, and to confirm that such particles are driven only by Brownian motion. While motor activity can be controlled *in vitro* by simply changing ATP concentration, altering the intracellular concentration of ATP *in vivo* affects a multitude of cellular processes ranging from active transport of intracellular molecules and ions to ubiquitous phosphorylation events, and thus would introduce confounding effects outside the realm of actomyosin contractility, making such an experimental approach unfeasible. Here, we take advantage of novel drugs to separately manipulate the motor and actin bundling activities of myosin II *in vivo*, and test directly Mizuno *et al*.'s hypothesis as to whether actomyosin structures produce nonthermal forces that can greatly enhance the motion of submicron beads in living cells [3] and, by extension, the motion of all submicron structures and organelles embedded in the cytoskeleton of cells. We find that actomyosin contractility does not significantly affect the motion of tracer particles, and thus that Einstein's equation is valid for describing the Brownian motion of our particles in living cells.

## Results and Discussion

### The bundling activity of myosin II does not affect bead motion

**Figure 1:**
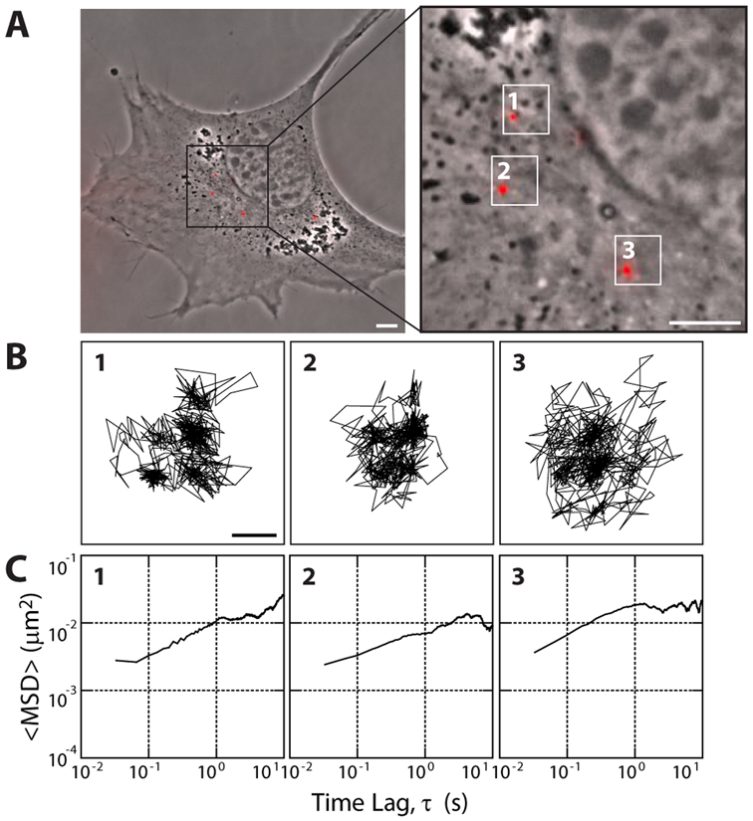
Multiple-particle tracking in Swiss 3T3 fibroblasts. A and B. Typical trajectories of 100 nm-diameter fluorescent beads that were bombarded in the cytoplasm of 3T3 fibroblasts. After overnight incubation, the beads were tracked with ∼10 nm spatial resolution and 33 ms temporal resolution for 20 s. Each slope discontinuity in (B) represents a 33 ms timestep. Scale bars, 20 µm (A) and 100 nm (B). C. Mean squared displacements (MSDs) of the beads 1–3 showed in panel A. The time lag-dependent MSDs showed a slope smaller than 1, indicative of sub-diffusive displacements within the cytoplasm.

**Figure 2:**
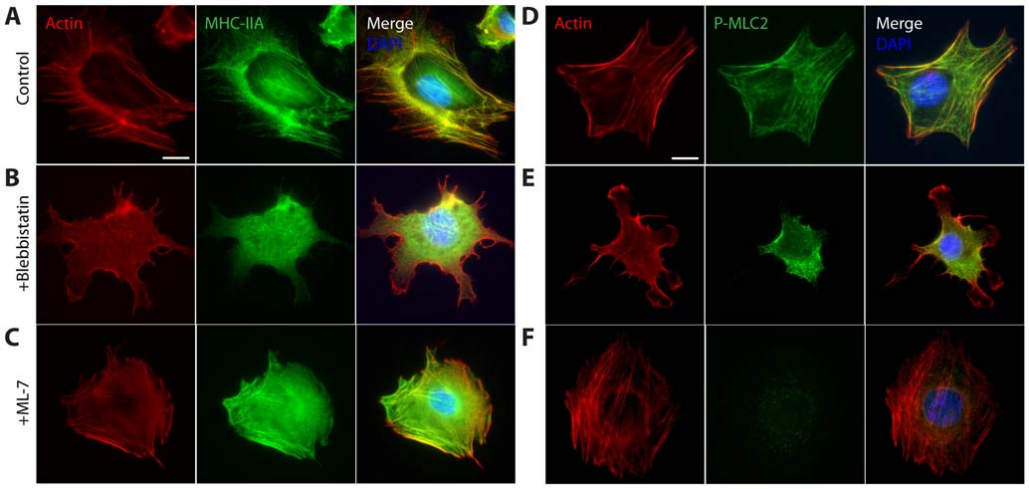
Cell morphology, actin, myosin, and phosphorylated-myosin organization following myosin inhibition. A–C. Typical morphology, actin filament organization (red), distribution of myosin heavy chain IIA (green), and nuclear DNA (blue) in control cells (A), cells treated with 25 µM myosin II inhibitor blebbistatin (B), and cells treated with 20 µM myosin light chain kinase inhibitor ML-7 (C). D–F. Typical morphology, actin filament organization (red), distribution of phosphorylated myosin light chain 2 (green), and nuclear DNA (blue) in control cells (D), cells treated with 25 µM myosin II inhibitor blebbistatin (E), and cells treated with 20 µM myosin light chain kinase inhibitor ML-7 (F). Scale bars, 20 µm.

One hundred-nm diameter polystyrene beads were directly bombarded into the cytoplasm of Swiss 3T3 fibroblasts, a type of cell routinely used to study and establish the functions of cytoskeletal proteins [15], [16]. Bombardment of beads as opposed to their passive engulfment circumvents the endocytic pathway that would encapsulate the beads in vesicles [11], which would then undergo microtubule motor-driven directed transport towards the nucleus [17]. Carboxylate-modified beads, used here, have been shown to bind roughly 1 actin molecule per microsphere when incubated with cell lysates, compared to 15 actin molecules for amine-modified beads [10]. Carboxylate-modified beads are thus assumed to behave as inert tracer particles with negligible levels of nonspecific binding. Additionally, bombardment was chosen over the traditional method of microinjection to allow for high-throughput monitoring of beads in cells. After overnight incubation of the cells, beads dispersed throughout the cytoplasm (Fig. 1). Particles at the extremities of the cell were not tracked to avoid tracking particles in height-limited regions. Importantly, bead size is significantly larger than the effective cytoskeletal mesh size (∼20–40 nm) [18]. Therefore, movements of these beads reflect the mesoscale viscoelastic properties of the cytoskeletal network in which they are embedded, not that of the lower viscosity of the interstitial fluid. Without assumptions about their mechanisms of transport, the beads' displacements were tracked using high-resolution video-microscopy (Fig. 1). The ensemble-averaged mean squared displacement (MSD) of beads in untreated fibroblasts, 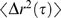, showed approximately diffusive behaviour at long time scales, 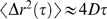, and the beginning of a plateau at short time scales, 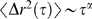 where 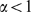 (Fig. 1) [19]. Untreated fibroblasts showed highly organized actin filament bundles terminated by vinculin-containing focal adhesions at the basal surface of the cells (Fig. 2, A and D, and Fig. S1). Both myosin II and activated (phosphorylated) myosin II, detected with antibodies against myosin heavy chain IIA and phospho-myosin light chain 2, respectively, co-localized with basal stress fibers and showed little staining in regions of the cytoplasm devoid of stress fibers (Fig. 2, A and D).

To investigate the role of myosin II in cellular stiffness, cells were treated with 25 µM blebbistatin, a drug that binds to the myosin-ADP-P_i_ complex with high affinity and interferes with the phosphate release process [20], [21], [22]. As a result, blebbistatin blocks myosin in a functionally inactive actin-detached state. After 45 min of blebbistatin treatment, the cellular organization of the actin filament network and the localization of phosphorylated myosin II in the cytoplasm were examined by immunofluorescence microscopy and compared to untreated cells. Blebbistatin eliminated both basal stress fibers (compare Fig. 2, A, B, D and E) and focal adhesions located at their ends (Fig. S1). A large fraction of myosin II molecules moved from the stress fibers to the cytoplasm as a result of blebbistatin treatment (Fig. 2B), remained phosphorylated and were distributed in a disorganized, punctate manner (Fig. 2E).

**Figure 3:**
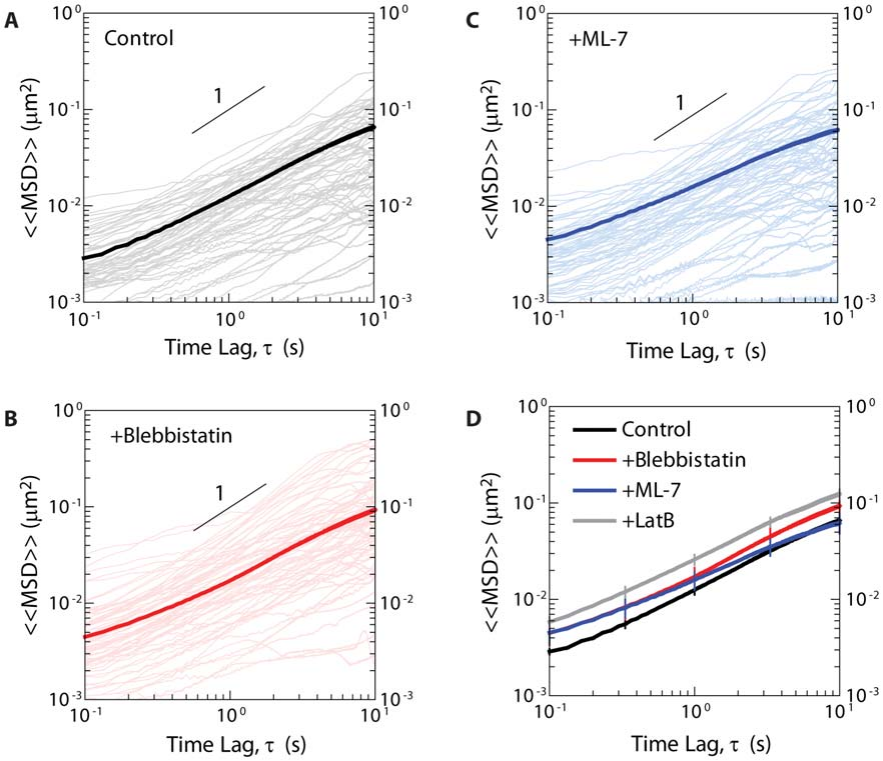
Role of myosin activity on the displacements of beads in the cytoplasm. A–C. Typical (light color) and ensemble-averaged MSDs (dark color) of beads embedded in the cytoplasm of control cells (A), cells treated with 25 µM blebbistatin (B), and cells treated with 20 µM ML-7 (C). D. Ensemble-averaged MSDs that are color-coded according to panels A–C. The time lag-dependent MSDs showed a slope smaller than unity, indicative of sub-diffusive displacements within the cytoplasm. At least 10 cells were probed per condition for a total of at least 30 cells (*n* = 3) and >200 particles.

We measured the MSD of beads embedded in blebbistatin-treated cells and found a slight, but statistically non-significant, increase in MSD magnitude relative to beads embedded in untreated cells (Fig. 3, A and B). MSD traces for individual particles reflect the heterogeneity of the cytoskeletal network, common to microrheological measurements [10]. The degree of heterogeneity, however, was not affected by blebbistatin treatment relative to untreated cells (Fig. 3, A and B). Note that although the degree of heterogeneity for all conditions spans roughly an order of magnitude, these data are highly reproducible. These results suggest that activated myosin II plays little or no significant role in modulating [3] the movements of the beads embedded in the cytoplasm of living cells, and thus indicate that the nonthermal forces generated by the bundling activity of myosin II do not overpower the thermal fluctuations responsible for bead movement inside living cells.

Although our previous studies have suggested actomyosin interactions as effectors of modulating cell stiffness [11], these studies were performed using butanedione 2-monoxime, or BDM, which has since proven to lack the specificity and potency as a nonmuscle myosin inhibitor as was originally presumed [23]. Furthermore, the aforementioned study probed the role of actin and myosin interactions in cells under shear, while the current study focuses strictly on quiescent cells.

### The motor activity of myosin II does not affect nanoparticle motion

To further test the effects of myosin II on bead movement without direct interference, we targeted an upstream activator of myosin II, myosin light chain kinase. Fibroblasts were treated with 20 µM ML-7, a myosin light chain kinase inhibitor that prevents specific phosphorylation of myosin II [24], [25]. In contrast to blebbistatin-treated cells, cells treated with ML-7 retained prominent actin filament bundles of length and width similar to those in untreated cells (Fig. 2, C and F). These bundles continued to contain myosin II molecules (Fig. 2C). Therefore, ML-7 treatment preserves the F-actin bundling activity of myosin. Nevertheless, the majority of myosin in basal actin filament bundles was de-phosphorylated with the remaining phosphorylated myosin molecules dispersed in the cytoplasm, evident in the very low phosphorylation levels seen in immunofluorescence staining (Fig. 2F). This small fraction of phosphorylated myosin exists presumably due to residual ROCK-mediated phosphorylation of myosin [26]. ML-7 treatment diminished the size of terminal focal adhesions relative to untreated cells (Fig. S1), suggesting a significant reduction in the tension within stress fibers, most likely due to the dephosphorylation of myosin [27]. Hence ML-7 treatment greatly reduced the tension-generating contractility of actomyosin structures in cells, while preserving myosin's ability to bundle actin filaments.

Surprisingly, in ML-7-treated cells, the time-dependence and magnitude of the mean squared displacement of beads were similar to those in control cells (Fig. 3, C and D). As with blebbistatin-treated fibroblasts, ML-7 treated cells did not show a drastic change in the degree of heterogeneity of individual particle MSDs relative to untreated cells (Fig. 3, A and C). As a positive control, we verified that the bulk disassembly of actin filaments in the cytoplasm using 1 µM latrunculin B increased the MSDs of beads (Fig. 3D). Together these results suggest that not only the bundling activity of myosin II, but also the motor activity of myosin II plays no significant role in driving the fluctuating movements of beads embedded in the cytoplasm of living cells. These results are in striking contrast with those obtained by Mizuno *et al*. using simpler, *in vitro* networks composed of purified actin and myosin II [3].

Previous studies have also investigated the role of actomyosin contractility on microrheological measurements, albeit with different methods that jeopardize the validity of their findings. In particular, Van Citters *et al.* used internal laser tracking microrheology (LTM) which studies the motion of endocytosed particles [28] as opposed to the inert particles embedded into the cytosol via bombardment used in this study. Endocytosed particles have been shown to move along microtubules with the help of microtubule motors such as kinesin [29]; therefore, movement of endocytosed beads is most likely driven not by thermal fluctuations, but rather by active microtubule motors at all time scales, even at short times scales where motion may appear Brownian. Studying endocytosed particles would therefore reveal an effective cellular stiffness along microtubules that is heavily dependent on instantaneous motor activity, whereas the study of our particles bombarded into the cytosol reveal a non-artifactual, actin-sensitive cellular stiffness. In fact, Van Citters *et al.* found that actin filament disruption had no effect on the functional form nor the amplitude of the cell interior's frequency-dependent rheological response, as probed by LTM. In direct contrast to this data, we see an increase in the ensemble-averaged MSD of latrunculin B-treated cells relative to untreated cells, indicating that our methodology is sensitive to actin assembly perturbations. Furthermore, as demonstrated previously with microinjected and endocytosed particles [10], we directly compared tracking data from bombarded and endocytosed particles, and found that indeed, the ensemble-averaged MSD of endocytosed particles was not only superdiffusive, but also much larger than that of subdiffusive bombarded particles (Fig. S2), providing further support that results obtained with endocytosed particles are skewed by active motion and ultimately unsuitable for assessing actomyosin contractility in living cells.

### MSD dependence on spatial postioning

Particles were also partitioned into perinuclear and lamellar regions to assess the spatial dependence of myosin-inhibiting drugs. As described previously [30], particles located within a circle, 30 µm in diameter, centered on the nucleus were deemed perinuclear, while particles outside of this region were deemed lamellar. We have previously shown that in mouse embryonic fibroblasts, particles located in a cell's lamellar region probe a stiffer environment than that of the perinuclear region, suggesting that lamellar particles are entangled in an actin network higher in density compared to that of the perinuclear region. Interestingly, in untreated 3T3 fibroblasts, the ensemble-averaged MSD from particles in lamellar regions did not differ drastically from that of particles in perinuclear regions (Fig. S3), and differences in ensemble-averaged MSD values at time lags τ = 0.1, 1, and 10 s between perinuclear and lamellar populations were not statistically significant. Furthermore, no statistically significant differences were detected between MSDs of control and drug-treated fibroblasts in either lamellar or perinuclear subpopulations (Fig. S3). These results suggest a weak dependence of particle MSD on spatial positioning in 3T3 fibroblasts and furthermore hints that spatial-dependent stiffnesses is cell-line dependent.

### Correlated motions of particle pairs

To further test the role of actomyosin contractility on particles embedded in the cellular cytoskeleton, we calculated the cross-correlation magnitudes of particle pairs in untreated, blebbistatin-treated, and ML-7-treated cells. When cells are in a non-contractile, or equilibrium, state, cross-correlations must be positive. During contractile events, however, cross-correlations can be positive, negative, or negligible over an established frequency range [3]. Mizuno *et al*. previously reported anticorrelated motions of bead pairs in reconstituted actomyosin networks, which they attributed to non-equilibrium, contractile/compressive gel deformations [3]. This result suggests that actomyosin contractility can be the dominant force on tracer particles, thereby invalidating the assumption that particle motion is strictly under the influence of Brownian motion.

**Figure 4:**
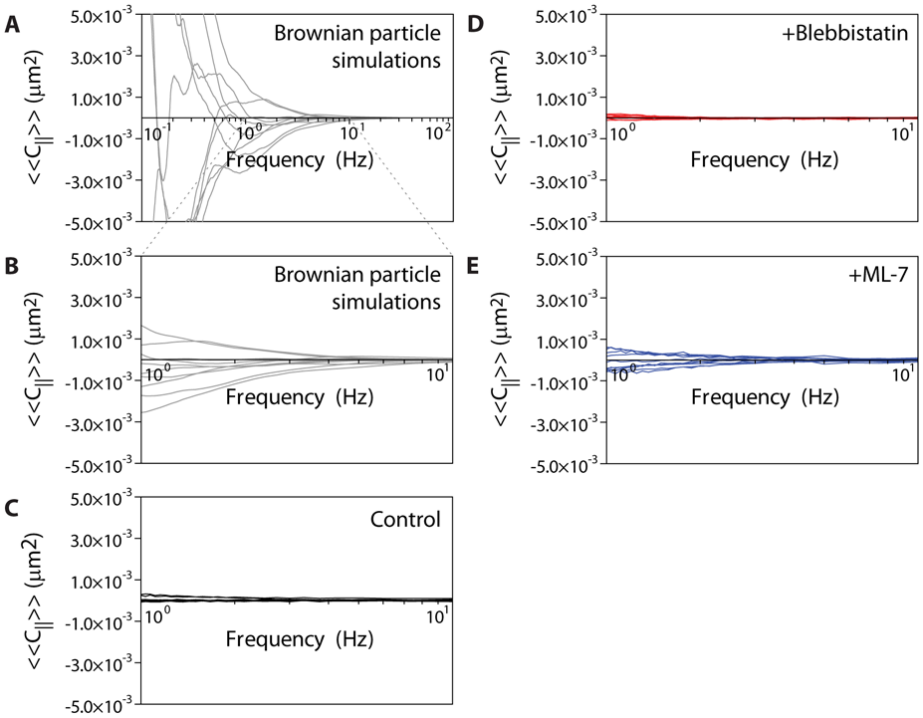
Cross-correlation magnitudes of simulated Brownian particles and particles in treated and untreated fibroblasts. A–B. Cross-correlation magnitudes of simulated Brownian particle pairs whose average diffusion coefficient is on the same order of magnitude as in untreated and drug-treated fibroblasts. Traces consistently above the x-axis represent positively correlated particle pairs, whereas traces consistently below the x-axis represent negatively correlated particle pairs. Since Brownian particle pairs were generated randomly and should not maintain any meaningful correlational data, these traces are the established “noise” threshold—magnitudes greater than Brownian pair traces are considered significant, while magnitudes lower than these tracers are considered uncorrelated over the frequency range presented. The data is redisplayed showing only the frequency range of interest, 1 to 10 Hz (B). C. Cross-correlation magnitudes of particle pairs in untreated fibroblasts. D. Cross-correlation magnitudes of particle pairs in blebbistatin-treated fibroblasts. E. Cross-correlation magnitudes of particle pairs in ML-7-treated fibroblasts. At least 10 different, representative particle pair traces are shown for each condition.

Based on the minimal effects that blebbistatin and ML-7 had on our microrheology measurements above, we hypothesized that, *in vivo*, such anticorrelated motion would not be detected. To test this hypothesis, we first simulated a population of Brownian particles whose time-averaged diffusion coefficient was on the same order of magnitude as that of particles in untreated, blebbistatin-treated, and ML-7-treated fibroblasts. The trajectories of these particles are purely random and thus should not display any positive or negative correlations. Therefore, we established any possible correlations between these particle pairs as our threshold of detecting meaningful correlations. Correlations would only be significant if their magnitudes over a defined frequency range were greater than those seen in simulated Brownian particles, whereas correlations whose magnitudes were less than those seen in simulated Brownian particles would be considered uncorrelated. Over the frequency range between 1 and 10 Hz, cross-correlations between particle pairs in all conditions were uncorrelated relative to Brownian particle simulations, suggesting that microrheology measurements are not affected by actomyosin contractile events (Fig. 4).

This discrepancy between *in vitro* and *in vivo* results suggests that the cytoplasmic environment studied here differs from the three-component model system of myosin II, actin filaments, and cross-linkers. The uncorrelated motion, as opposed to positively correlated motion, seen here at the lower frequencies measured, also reinforces the notion that global cell movements do not interfere with particle tracking measurements.

### The mechanical architecture of adherent cells

Our results suggest an explanation for the apparently contradictory results obtained in live cells and in reconstituted actin/myosin networks [3]. Myosin II is located almost exclusively in contractile stress fibers in the basal and apical regions of adherent cells (Fig. 2, A and D), from which beads are excluded. We found that the deactivation of the motor activity of myosin II structurally and functionally only affects the stress fibers, not other actin structures in the cytoplasm and does not significantly affect the motion of beads embedded in the cytoplasm. Indeed our results show that the spontaneous motion of the beads is largely unaffected by the bundling and contractile activities of actomyosin stress fibers, both in perinuclear and lamellar bead populations. Nevertheless, myosin II is the main motor protein driving the contraction of the actin filament cytoskeleton, not other myosins (such as myosin I, myosin V, etc.) or microtubule-bound motor proteins such as kinesin or dynein, which are mainly involved in vesicular trafficking and filopodial formation in interphase cells [31]. Stress fibers are contractile and are responsible, for instance, for the deformation of the extracellular matrix during motility events. In contrast, in the body of the cell, short actin filaments form a dense meshwork that is dynamically crosslinked by F-actin binding proteins such as filamin and α-actinin [14]. The fact that the organization of stress fibers depends critically on myosin activity and that the organization of cytoplasmic actin structures in the body of the cell does not depend on myosin activity suggest that the actin filament meshwork in the body of the cell and the contractile stress fibers at the periphery of the cell (i.e. the apical and ventral surfaces as well as the leading and trailing edges of the cell) are mechanically decoupled. Thus, our results do not explicitly contradict those of Mizuno *et al.*; it is plausible that reconstituted actin networks better mimic actin behavior at the cell cortex where motor activity is a dominant factor, whereas *in vivo* microrheology probes the cytoplasmic actin network decoupled from stress fibers.

Our results strongly suggest that particle-tracking microrheology analysis correctly assumes that the fluctuating movements of probing beads are driven strictly by thermal forces, not by nonthermal forces that could have been induced by tension-generating myosin. While it remains possible that the random forces exerted on particles could be due to microtubule motion, it is difficult to address this question since microtubule depolymerization can alter Rho GTPase activity [32], and thus indirectly affect actin dynamics and the movements of our tracer particles. Nevertheless, the fact that our particle pairs do not show significant positive correlations suggests that microtubule movement does not affect the movement of our tracer particles.

**Figure 5:**
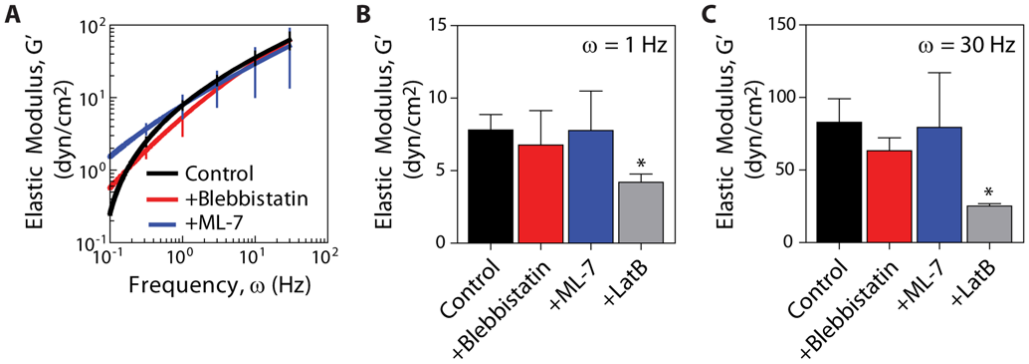
Role of myosin activity in intracellular microrheology. A. Averaged frequency-dependent elastic modulus of the cytoplasm of control cells (black), cells treated with blebbistatin (red), and cells treated with ML-7 (blue). B and C. Averaged cytoplasmic elastic moduli at frequencies of 1 Hz (B) and 30 Hz (C). At least 10 cells were probed per condition for a total of at least 30 cells (*n* = 3) and >200 particles.

Since the MSD profiles are largely independent of F-actin bundling and motor activities of myosin, we found that the microrheology analysis of the MSDs gave rise to similar averaged viscous and elastic moduli in the three tested conditions at both low and high frequencies, or long and short time scales, respectively (Fig. 5, A–C). Our particle-tracking microrheology shows that the cytoplasm of living cells behaves as a viscoelastic liquid at long time scales. Indeed, the MSDs of 100 nm-diameter beads larger than the average mesh size of the cytoskeleton (∼20–40 nm [18]) are almost proportional to time lag, albeit with a slope that is much lower than MSDs of the same beads in water (Fig. 3D). The cytoplasmic shear viscosity is approximately 24 Poise, or 2.4 Pa·s, about 2400 times that of water, which is close to the viscosity of the interstitial liquid of the cell. The fact that the cytoplasm is soft and behaves more like a liquid than a solid at long time scales seems to contradict AFM measurements of cell mechanics, which would suggest that the subcellular milieu is extremely stiff, with a modulus of the order of kPa [33], [34], [35], hundreds of times higher than the elastic modulus extracted from the particle tracking microrheology analysis performed here.

### AFM and particle tracking microrheology are not contradictory

**Figure 6:**
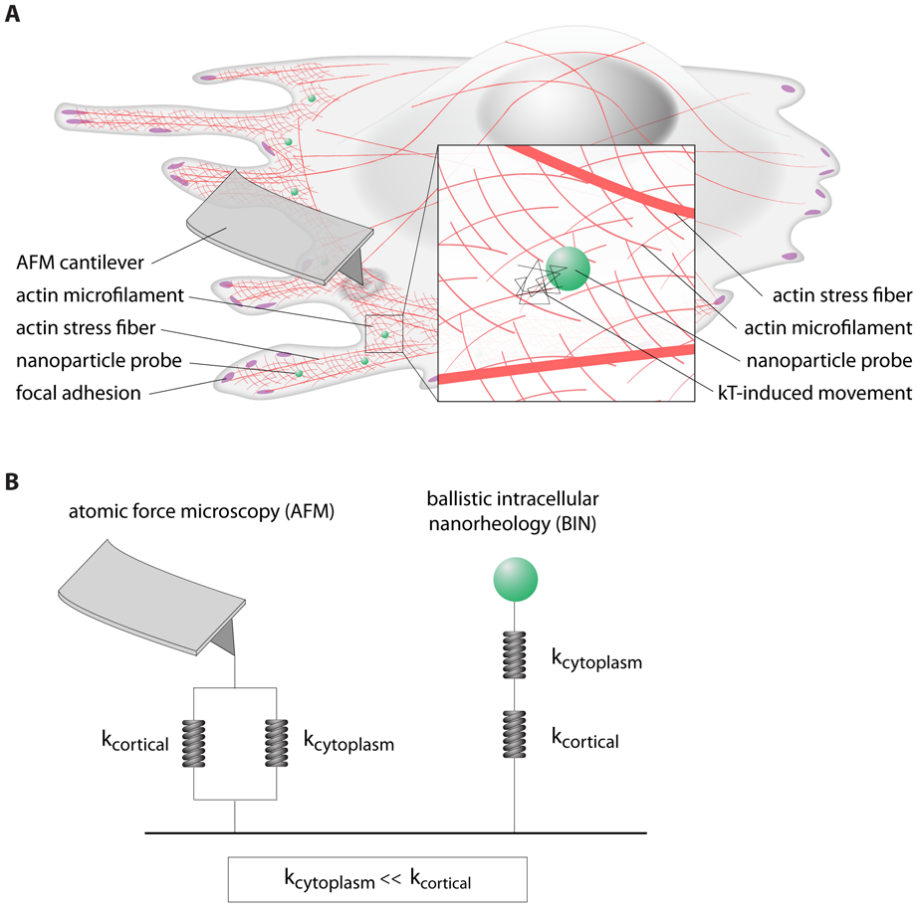
Mechanical architecture of adherent cells. A. The cytoskeleton of adherent cells is composed of stiff contractile actomyosin structures located in the cortex of the cell, including the basal and apical surfaces of the lamella and the top of the nucleus, which envelop a soft actin filament network devoid of stress fibers. B. Simple model describing how cell mechanics is measured differently by AFM and particle tracking microrheology. AFM measures the combined response of stiff contractile stress fibers and the soft actin network weaved within it. This is equivalent to a soft and a stiff spring in parallel. Particle tracking microrheology only measures the soft actin filament network in the body of the cell that surrounds each bead, which is devoid of stress fibers. This is equivalent to a soft and a stiff spring in series.

Results from immunofluorescence microscopy suggest that traditional AFM measurements and microrheology analysis are not necessarily incompatible. It is plausible that these two techniques simply probe different regions of the cell which are mechanically engaged differently depending on their mechanical input. AFM subjects the cell surface to preset deformations and is, therefore, greatly sensitive to the cortical structures of the cells. These structures contain stiff actin stress fibers that interdigitate the actin meshwork both on top of the nucleus and at the basal and dorsal surfaces of the thin lamella region away from the nucleus (Fig. 6A) [36].

In contrast, in particle-tracking measurements, probing beads are mostly lodged in cytoplasmic regions that are devoid of contractile stress fibers. The beads are sterically excluded from the stress fibers above the nucleus and at the cell cortex, the regions accessible by AFM (see more below). High-resolution tracking of beads in the cytoplasm suggests that, from a rheological point of view, the body of the cell is liquid-like at time scales as short as a few seconds, presumably because this region of the cell contains short filaments. Their disassembly by latrunculin B causes cytoplasmic softening (Fig. 5C). Hence, the only solid-like structure that can maintain the shape of cells is not the dense network of actin filaments in the body of the cell, but the nucleus and the rather stiff (yet dynamic) stress fibers at the cell's basal surface as well as the contractile stress fibers in the perinuclear actin cap, which wraps around the nucleus. Hence, at long time scales, the mechanical architecture of an adherent cell is composed of thick contractile structures at the basal and apical surfaces of the cell cortex and a highly viscous polymer liquid located both in the body of the cell and in the space between stress fibers. At short time scales, both cytoplasmic and cortical actin structures can contribute to the elasticity of the cell.

### Contractile actin network in the cortex *vs*. viscoelastic actin network in the cytoplasm

The overall mechanical properties of an adherent cell, as probed by AFM and particle tracking microrheology, can be modelled as springs in parallel and in series, respectively (Fig. 6, A and B; here we ignore viscous effects). An AFM tip (or large bead attached to the tip) imposes a defined deformation of the cell surface, which engages both the soft cytoplasmic region in the body of the cell and the stiff cortical region containing discrete stress fibers networked at the apical and basal surfaces of the lamella and on top of the nucleus. From a rheological standpoint, the response of the cell from deformation by an AFM tip is equivalent to two elastic springs in parallel (Fig. 6B): one with a low spring constant, 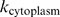, and one with a high spring constant, 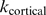. In this case, the resistance to the AFM-imposed deformation 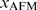 of the cell is dominated by the stiff cell cortex which contains both stress fibers and the dense actin meshwork, not the actin network in the body of the cell, such that 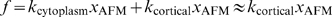, where 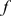 is the AFM force necessary to generate the cellular deformation 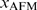. In contrast, a bead embedded in the actin meshwork of the cytoplasm (and excluded from the stress fibers) will impose a fluctuating thermally-excited force on its surrounding soft cytoplasmic milieu, which may (or may not) be physically connected to distal stiff stress fibers in the cell cortex (Fig. 6, A and B). This is equivalent to two springs in series (Fig. 6B). In this case, the total deformation, 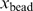, imposed by the fluctuating bead on its cytoplasmic surroundings is dominated by the soft spring, i.e. the cytoplasm, 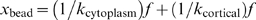 and 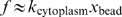. Therefore, the fluctuating force 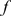 on the bead mechanically engages only the soft cytoplasm around the bead, not the stiff stress fibers in cell cortex (Fig. 6B).

In summary, our results: (i) resolve the role of myosin and contractile actomyosin structures in cell mechanics, (ii) support the validity of particle tracking microrheology measurements, (iii) reveal the biphasic mechanical architecture of adherent cells: stiff stress fibers interdigitating in the cell cortex (above the nucleus and in the lamella) and a soft actin meshwork in the body of the cell, and (iv) reconcile seemingly contradictory AFM and microrheology measurements, resolving a long-standing issue in the field of cell mechanics.

## Materials and Methods

### Cell culture

Swiss 3T3 fibroblasts (ATCC, Manassas, VA) were cultured in DMEM supplemented with 10% bovine calf serum (BCS, ATCC), 100 U penicillin and 100 µg streptomycin (Sigma, St. Louis, MO) and maintained at 37°C in a humidified, 5% CO_2_ environment. Cells were passaged every 3–4 days. For immunofluorescence microscopy, cells were seeded at ∼2×10^3^ cells/ml on 35-mm glass bottom dishes pre-coated with collagen (MatTek, Ashland, MA). For ballistic nanoparticle injection, cells were seeded at ∼1×10^4^ cells/ml on 35-mm cell culture dishes (Corning, Corning, NY). Following bombardment and recovery, cells were then seeded at ∼2×10^3^ cells/ml on 35-mm glass bottom collagen-coated dishes.

### Drug treatments

The myosin light chain kinase inhibitor ML-7 (Sigma), the nonmuscle myosin II inhibitor (-) blebbistatin (Sigma), and the F-actin disassembly drug latrunculin B (Sigma) were diluted from stock using culture media. ML-7 was used at a final concentration of 20 µM. Blebbistatin was used at a final concentration of 25 µM. Latrunculin B was used at a final concentration of 1 µM. Cells were incubated with either ML-7 or blebbistatin for 45 min, or latrunculin B for 30 min, prior to fixation or particle tracking. Drug concentrations were maintained during experiments to avoid recovery.

### Immunofluorescence microscopy

Cells plated on glass bottom collagen-coated dishes were fixed with 3.7% formaldehyde for 30 min, washed with 1x phosphate-buffered saline (PBS) at room temperature (RT) and permeabilized with 0.1% Triton X-100 for 10 min. PBS supplemented with BCS (10%) was used to block nonspecific binding, after which cells were treated with primary and secondary antibodies, respectively, at proper dilutions for 1 hour each at RT. For myosin heavy chain IIA staining, cells were incubated with a rabbit anti-nonmuscle myosin II heavy chain A polyclonal antibody (Covance, Princeton, NJ), used at 1∶250 dilution, and subsequently incubated in Alexa Fluor 488 goat anti-rabbit antibody (Invitrogen) at 1∶200 dilution. For phosphorylated-myosin light chain II staining, cells were incubated with a monoclonal mouse anti-phospho-myosin light chain II antibody (Cell Signaling Technology, Danvers, MA) at 1∶50 dilution, and subsequently incubated in Alexa Fluor 488 goat anti-mouse antibody (Invitrogen, Carlsbad, CA) at 1∶200 dilution. For supplemental staining, mouse anti-vinculin monoclonal antibody (Sigma) was used at 1∶40 dilution, after which cells were incubated with Alexa Fluor 488 goat anti-mouse (Invitrogen) at 1∶200 dilution. Actin and nuclear DNA were stained during secondary treatment using Alexa Fluor 568 phalloidin (Invitrogen) at 1∶40 dilution and 300 nM DAPI, respectively. Cells were then cured in ProLong Gold antifade reagent (Sigma) and then covered with a cover slip prior to visualization. Fluorescent micrographs were collected using a Cascade 1K CCD camera (Roper Scientific, Tucson, AZ) mounted on a Nikon TE2000E microscope with a 60× Plan Fluor lens (N.A. 1.4, Nikon, Melville, NY) controlled by Metavue (Universal Imaging, West Chester, PA). Images were digitally overlayed using Metamorph (Universal Imaging).

### Particle tracking and cell microrheology

The micro-mechanical properties of the cytoplasm were measured using ballistic intracellular nanorheology (BIN), as described previously [11]. 100 nm-diameter fluorescent polystyrene nanoparticles (Invitrogen) were ballistically injected in the cytoplasm of cells using a Biolistic PDS-1000/HE particle delivery system (Bio-Rad, Richmond, CA). Nanoparticles were coated on macrocarriers and allowed to dry for 2 h. 900 psi rupture disks were used. Cells were repeatedly washed post-bombardment to eliminate endocytosis of the nanoparticles, thus avoiding the possibility of convective, vesicular transport of the particles within the cell. We verified that none of the probed nanoparticles underwent directed motion. Cells were given several hours to recover post-bombardment before embedded nanoparticles were tracked with high spatial and temporal resolutions using high-magnification fluorescence microscopy (60×). An optimized region of interest (ROI) surrounding target particles was generated using Metavue software for recording particle videos acquired with 3×3 binning at 30 frames per second for 20 seconds, for a total of 600 frames per movie. Movies capturing the Brownian motion of the fluorescent nanoparticles were collected and analyzed first using Metamorph software; custom software was subsequently used to obtain rheological parameters describing the viscoelastic properties of the cytoplasm [37]. At least 200 different nanoparticles were tracked per condition.

Particles at the extremities of the cell were not tracked to avoid tracking particles in height-limited regions. After injection and incubation overnight, nanoparticles dispersed uniformly throughout the cytoplasm. For spatial analysis of particle tracking data (Fig. S3), beads were pooled into one of two regions. Beads located within a circle, 30 µm in diameter, centered on the nucleus, were deemed perinuclear, while beads outside of this region were deemed lamellar. Since neither the perinuclear nor lamellar region is overrepresented in each nanoparticle population, the differences in elasticity presented in Figures 3 and 5 represent global changes in cytoskeletal stiffness as opposed to location-specific stiffnesses. The time-averaged MSD, 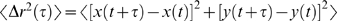, where 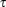 is the timescale and 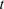 is the elapsed time, was calculated from the trajectory of the light intensity-weighted centroid of each nanoparticle. Previous studies have examined the effects of size and surface chemistry of the nanoparticles in cells [10], [38]. It is important to note that each movie is much shorter than the characteristic timescale of cell migration. Given the organization of the actin network in the cytoplasm [39], we assumed that the particles were not biased towards movement in any particular direction. Thus, we assumed that the time-averaged movements of the nanoparticles in the *x-*, *y-*, and *z-*directions were identical, such that 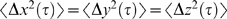 so that 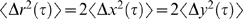 [40]. This suggests that, for the short times of movie capture, the cytoplasm around each nanoparticle can be considered isotopic, i.e., it has the same physical properties in the *x-*, *y-*, and *z-*directions.

By tracking the centroid displacements of the nanoparticles of interest, we can reach a subpixel spatial resolution well below the 200 nm limiting size resolution of a light microscope. A small displacement of the nanoparticle will induce a displacement of the centroid of the light-intensity profile of the diffraction-limited image of each nanoparticle, which is readily detected with subpixel resolution. As reported previously, by tethering nanoparticles to glass coverslips to “immobilize” the particles, we have determined the real displacement resolution of our microscopy/particle-tracking system to be ∼10 nm.

The mean elasticity of the cytoplasm is calculated from the ensemble-averaged MSD, as described [41]. Briefly, the ensemble-averaged MSD of the nanoparticles is related to the complex viscoelastic modulus using the following equation [41], [42],
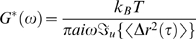
where 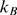 is Boltzmann's constant, 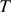 is the absolute temperature of the cell, 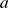 is the radius of the nanoparticles, and 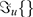 is the Fourier transform of 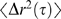, the time lag-dependent ensemble-averaged MSD. The above equation can be solved analytically allowing the frequency-dependent elastic modulus to be calculated algebraically using the relationship 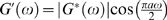, where
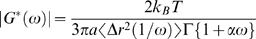



Here 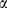 is the local logarithmic slope of 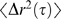 at the frequency of interest and 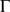 is the gamma function. The elastic modulus, 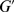, describes the propensity of a complex fluid to store energy. A cross-linked filamentous structure, such as a reconstituted F-actin network [43] or the cytoplasm [44], behaves like a solid elastic gel at high rates of shear (high frequencies 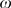) because the filaments do not have the time to relax during shear, and like a liquid at low rates of shear.

### Correlated motion of simulated Brownian and fibroblast particle pairs

Two-dimensional Brownian particles were simulated using the following equation for *x-* and *y-*coordinates at each time step,
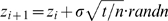
where 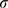 is a noise parameter that correlates with diffusivity, *t* is the maximum time of simulation in seconds, 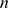 is the number of steps, and 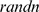 represents a pseudorandom value drawn from the standard normal distribution. The noise parameter 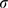 was adjusted such that the time-averaged diffusion coefficient of a population of Brownian particles was on the same order of magnitude as the time-averaged diffusion coefficient of all populations of particles in both untreated and treated fibroblasts.

Cross-correlation magnitudes of particle pairs were computed directly from particle trajectories, acquired through Metamorph software as described above. Custom software was written to determine the frequency-dependent correlated motion of a particle pair, calculated using the following equation,
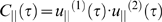
where 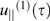 and 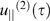 represent the dot products of the vectors created by the particles displacements at a particular time lag and the normalized vector connecting the initial coordinates of the two particles for each particle, respectively. Cross-correlation magnitudes were calculated at each time lag, ensemble-averaged, and plotted as a function of frequency.

### Statistics

Immunofluorescence microscopy and ballistic intracellular nanorheology were assessed in at least 3 independent experiments for all cell conditions studied. Statistical analysis was performed and mean values and standard error of measurement (SEM) were calculated and plotted using Graphpad Prism (Graphpad Software, San Diego, CA). Two-tailed unpaired *t* tests were conducted to determine significance of subdiffusive populations among different conditions (no significant differences detected). Significance was assessed using the standard Michelin Guide scale.

## Supporting Information

Figure S1 Focal adhesion localization following myosin inhibition. A–C. Typical morphology and focal adhesion localization assessed by vinculin staining in control cells (A), cells treated with 25 µM myosin II inhibitor blebbistatin (B), and cells treated with 20 µM myosin light chain kinase inhibitor ML-7 (C). Scale bar, 20 µm.
(1.11 MB PDF)

Figure S2 Movements of endocytosed particles versus bombarded particles. Ensemble-averaged MSDs of bombarded particles (black) and endocytosed particles (red) in untreated 3T3 fibroblasts. Bombarded particles showed a subdiffusive ensemble-averaged MSD with a slope less than 1 at all time scales, while endocytosed particles showed a superdiffusive ensemble-averaged MSD with a slope greater than 1 over the majority of time scales probed. At least 10 cells were probed per condition with >100 particles per condition.
(0.27 MB PDF)

Figure S3 Spatial analysis of particle tracking data. A. Representative 3T3 fibroblast with white circle delineating perinuclear from lamellar region. The perinuclear region was defined as the area encompassing a circle, 30 µm in diameter, centered on the nucleus. The lamellar region includes the cell area outside the perinuclear region. Particles were tracked, marked as either perinuclear or lamellar, and ensemble-averaged with particles located in the same respective region of the cell. Scale bar, 10 µm. B. Ensemble-averaged MSDs of perinuclear (light gray), lamellar (dark gray), and unpartitioned, or all, (black) particles embedded in the cytoplasm of control cells. C–D. Ensemble-averaged MSDs of only perinuclear particles embedded in the cytoplasm of control cells (black), cells treated with 25 µM blebbistation (red), and cells treated with 20 µM ML-7 (blue). Differences between control and drug-treated conditions were not statistically significant for all time points shown. E–F. Ensemble-averaged MSDs of only lamellar particles embedded in the cytoplasm of control cells (black), cells treated with 25 µM blebbistation (red), and cells treated with 20 µM ML-7 (blue). Differences between control and drug-treated conditions were not statistically significant for all time points shown. At least 20 cells were probed per condition with at least 20 particles per condition.
(1.92 MB PDF)
