# Supplementary figures and images for: Resolving the Role of Actoymyosin Contractility in Cell Microrheology
Source: PLoS One. 2009 Sep 16;4(9):e7054. doi: 10.1371/journal.pone.0007054 (PMC2737638; doi:10.1371/journal.pone.0007054)

**FIGURE S1**

A

Control

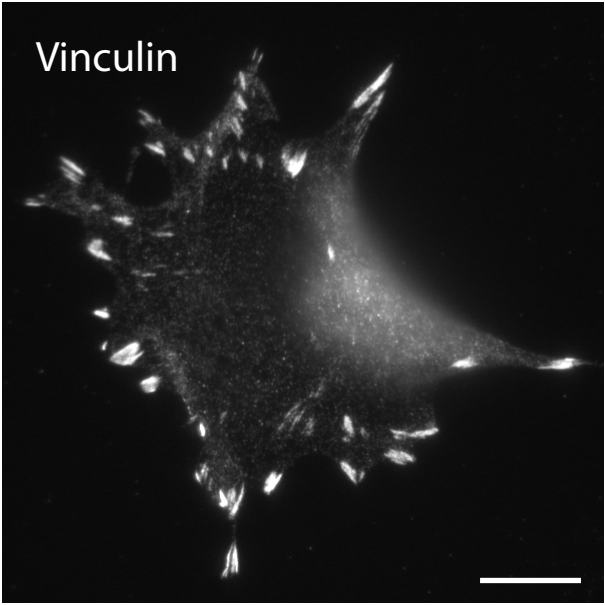

B

+Blebbistatin

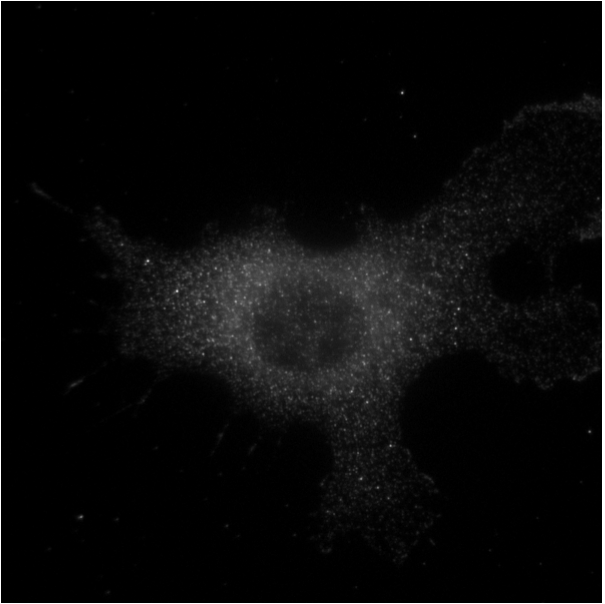

C

+ML-7

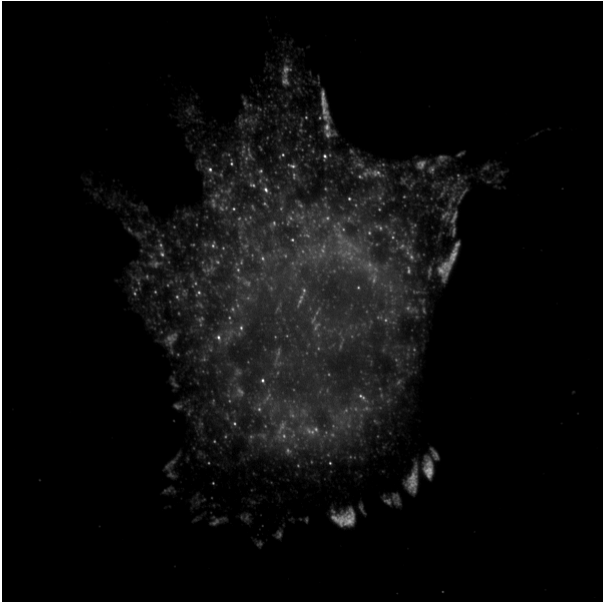

Supplement: Figure S1 — Focal adhesion localization following myosin inhibition. A–C. Typical morphology and focal adhesion localization assessed by vinculin staining in control cells (A), cells treated with 25 µM myosin II inhibitor blebbistatin (B), and cells treated with 20 µM myosin light chain kinase inhibitor ML-7 (C). Scale bar, 20 µm. (1.11 MB PDF) [file pone.0007054.s001.pdf]

# FIGURE S2

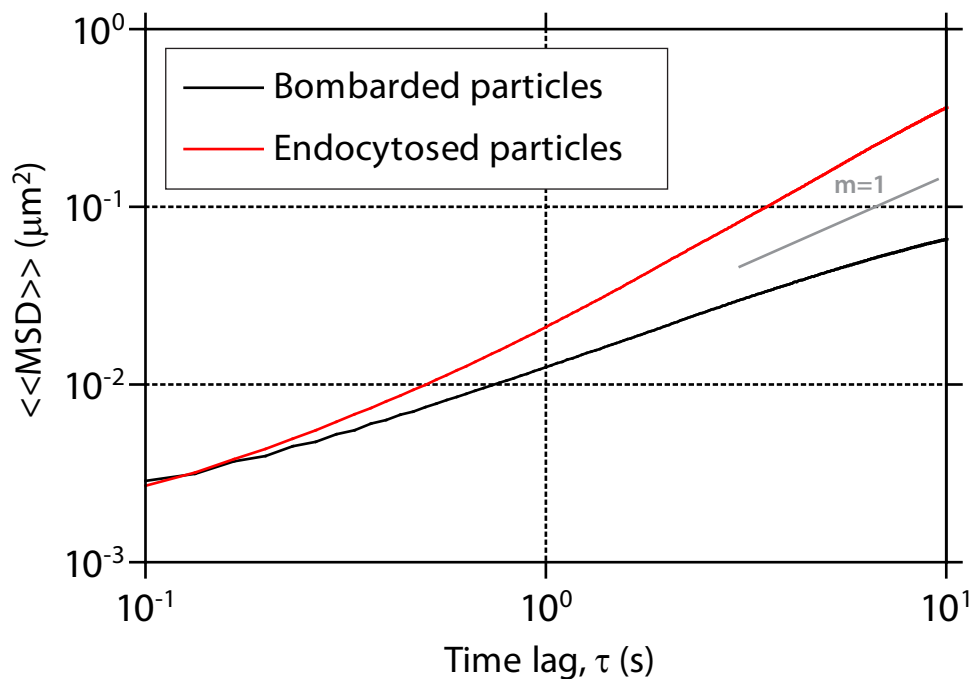

Supplement: Figure S2 — Movements of endocytosed particles versus bombarded particles. Ensemble-averaged MSDs of bombarded particles (black) and endocytosed particles (red) in untreated 3T3 fibroblasts. Bombarded particles showed a subdiffusive ensemble-averaged MSD with a slope less than 1 at all time scales, while endocytosed particles showed a superdiffusive ensemble-averaged MSD with a slope greater than 1 over the majority of time scales probed. At least 10 cells were probed per condition with >100 particles per condition. (0.27 MB PDF) [file pone.0007054.s002.pdf]

# FIGURE S3

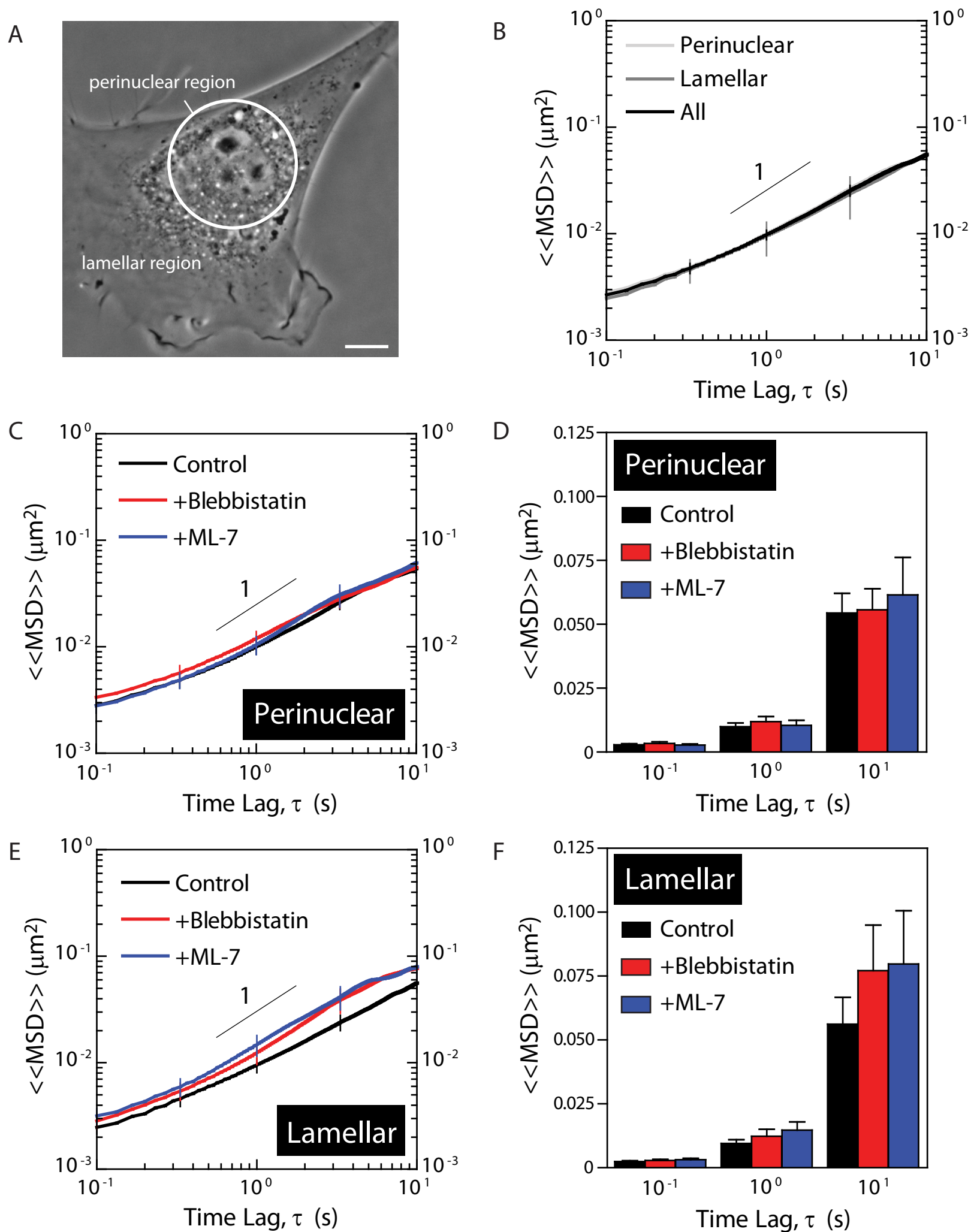

Supplement: Figure S3 — Spatial analysis of particle tracking data. A. Representative 3T3 fibroblast with white circle delineating perinuclear from lamellar region. The perinuclear region was defined as the area encompassing a circle, 30 µm in diameter, centered on the nucleus. The lamellar region includes the cell area outside the perinuclear region. Particles were tracked, marked as either perinuclear or lamellar, and ensemble-averaged with particles located in the same respective region of the cell. Scale bar, 10 µm. B. Ensemble-averaged MSDs of perinuclear (light gray), lamellar (dark gray), and unpartitioned, or all, (black) particles embedded in the cytoplasm of control cells. C–D. Ensemble-averaged MSDs of only perinuclear particles embedded in the cytoplasm of control cells (black), cells treated with 25 µM blebbistation (red), and cells treated with 20 µM ML-7 (blue). Differences between control and drug-treated conditions were not statistically significant for all time points shown. E–F. Ensemble-averaged MSDs of only lamellar particles embedded in the cytoplasm of control cells (black), cells treated with 25 µM blebbistation (red), and cells treated with 20 µM ML-7 (blue). Differences between control and drug-treated conditions were not statistically significant for all time points shown. At least 20 cells were probed per condition with at least 20 particles per condition. (1.92 MB PDF) [file pone.0007054.s003.pdf]
